# Construction and validation of a seven-gene signature for predicting overall survival in patients with kidney renal clear cell carcinoma via an integrated bioinformatics analysis

**DOI:** 10.1080/19768354.2020.1760932

**Published:** 2020-05-12

**Authors:** Huiming Jiang, Haibin Chen, Nanhui Chen

**Affiliations:** a Department of Urology, Meizhou People’s Hospital (Huangtang Hospital), Guangdong Provincial Key Laboratory of Precision Medicine and Clinical Translational Research of Hakka Population, Meizhou, People’s Republic of China; b Department of Histology and Embryology, Shantou University Medical College, Shantou, People’s Republic of China

**Keywords:** Kidney renal clear cell carcinoma, TCGA, ICGC, gene signature, prognosis

## Abstract

Kidney renal clear cell carcinoma (KIRC) remains a significant challenge worldwide because of its poor prognosis and high mortality rate, and accurate prognostic gene signatures are urgently required for individual therapy. This study aimed to construct and validate a seven-gene signature for predicting overall survival (OS) in patients with KIRC. The mRNA expression profile and clinical data of patients with KIRC were obtained from The Cancer Genome Atlas (TCGA) and International Cancer Genome Consortium (ICGC). Prognosis-associated genes were identified, and a prognostic gene signature was constructed. Then, the prognostic efficiency of the gene signature was assessed. The results obtained using data from the TCGA were validated using those from the ICGC and other online databases. Gene set enrichment analyses (GSEA) were performed to explore potential molecular mechanisms. A seven-gene signature (PODXL, SLC16A12, ZIC2, ATP2B3, KRT75, C20orf141, and CHGA) was constructed, and it was found to be effective in classifying KIRC patients into high- and low-risk groups, with significantly different survival based on the TCGA and ICGC validation data set. Cox regression analysis revealed that the seven-gene signature had an independent prognostic value. Then, we established a nomogram, including the seven-gene signature, which had a significant clinical net benefit. Interestingly, the seven-gene signature had a good performance in distinguishing KIRC from normal tissues. GSEA revealed that several oncological signatures and GO terms were enriched. This study developed a novel seven-gene signature and nomogram for predicting the OS of patients with KIRC, which may be helpful for clinicians in establishing individualized treatments.

## Introduction

Kidney cancer is a complex disease that encompasses different types of tumors. Kidney renal clear cell carcinoma (KIRC) is the most common pathological subtype, which accounts for about 70%–75% of kidney cancers (Shuch et al. 2015; Hakimi et al. 2019). With the increasing incidence and mortality in recent years, KIRC remains a significant challenge worldwide (Gray and Harris 2019). According to the recent cancer statistic report, the number of newly diagnosed cases in the United States has increased to 73,820 in 2019, and nearly 14,770 deaths were recorded (Siegel et al. 2019). In approximately 20% of KIRC patients, the condition progresses to advanced stages after diagnosis, and the 5-year overall survival (OS) rate of patients with metastatic cancer is less than 10% (Mitchell et al. 2018). Thus, prognostic models, which can accurately identify high-risk patients, are urgently needed.

Conventional models usually utilize clinicopathological parameters, including TNM stage and nuclear grade, in predicting the survival of patients with KIRC (Lam et al. 2008). However, these parameters are not highly accurate due to the significant heterogeneity of KIRC. The predictive ability of the conventional models is far from satisfactory. In recent years, with the rapid development of high-throughput sequencing techniques, several studies have shown that gene signatures at the mRNA level have a great potential in predicting the survival of patients with KIRC (Zhan et al. 2015; Chen, Luo et al. 2019; Hu et al. 2019; Pan et al. 2019; Wu et al. 2019; Zeng et al. 2019). Zhan et al. established a five gene signature (CKAP4, ISPD, MAN2A2, OTOF, and SLC40A1) for predicting the prognosis of KIRC using data from the Cancer Genome Atlas (TCGA) (Zhan et al. 2015). Moreover, Hu et al. constructed a seven-gene signature (BID, CCNF, DLX4, F AM72D, PYCR1, RUNX1, and TRIP13) for predicting the survival of patients with KIRC using data from the TCGA (Hu et al. 2019). However, the gene signatures were only based on TCGA data set, and they were not validated using other external data in most studies. Thus, only few of these signatures are used clinically, and more accurate and practical risk gene models for predicting the survival of KIRC must be developed. This study aimed to identify a prognostic gene signature based on information from several online databases. Further, its predictive ability was compared with that of other gene signatures.

## Materials and methods

### Data extraction

We downloaded the level 3 mRNA expression profiles and clinical data of 538 KIRC patients and 72 normal patients from the TCGA database (https://portal.gdc.cancer.gov/) and 91 KIRC patients from The International Cancer Genome Consortium (ICGC) database (https://icgc.org/).

### Identification of differentially expressed genes in KIRC

Normalization, log2 transformation, and differentially expressed gene (DEG) analysis was conducted using the R package DESeq2 (Love et al. 2014). Genes with a *P* value < 0.05 and |log2 fold change (FC)| > 1 were considered as DEGs.

### Construction of the prognostic gene signature

Univariate Cox regression analysis was performed to identify prognostic genes, and a *P* value < 0.001 was considered statistically significant. Then, the patients with a follow-up period longer than 1 month were randomly divided into training set and testing set. Lasso penalized Cox regression analysis was conducted to construct a prognostic gene signature, shown as risk score = (coefficient_gene1_ × expression level of gene1) + (coefficient_gene2 _×  expression level of gene2) + ⋯ + (coefficient_genen_ × expression level of genen) (Tibshirani 1997). The R package survival and survminer were used to investigate the optimal cutoff value of the risk score and establish the Kaplan–Meier survival curve (Diboun et al. 2006). The patients were classified into high- and low-risk groups based on the cutoff value. The R package survivalROC was used to investigate the prognostic value of the gene signature (Heagerty et al. 2000).

### External validation of the prognostic gene signature and gene change

The risk score of each patient in the ICGC cohort was calculated with the same prognostic gene signature. Then, ROC and Kaplan–Meier analyses were performed to validate the predictive ability of the gene signature. The expression of the genes in the gene signature was also further validated using information from the TIMER database (https://cistrome.shinyapps.io/timer/) and Oncomine database (https://www.oncomine.org/). In addition, the genetic alterations were investigated in the cBioportal for Cancer Genomics (https://www.cbioportal.org/).

### Independent prognostic role of the gene signature

Univariate and multivariate Cox regression and stratified analyses were performed to explore the independent prognostic role of the gene signature.

### Establishing and validating a predictive nomogram

All independent prognostic factors identified in the multivariate Cox regression analysis were used to construct a nomogram. Calibration and discrimination were performed to assess the nomogram with the calibration plot and concordance index (C-index), respectively. Subsequently, the models that included only one independent prognostic factor were compared with the combined model with all independent prognostic factors using the ROC curve, C-index and decision curve analyses (DCA) (Vickers and Elkin 2006).

### Differentiating capacity of the prognostic gene signature

We explored the capacity of the prognostic gene signature in distinguishing KIRC from normal tissues using boxplot and ROC curve.

### Gene set enrichment analyses

To further explore the underlying molecular mechanisms, gene set enrichment analyses (GSEA) were performed in H (Hallmark gene sets), C2 (KEGG), C5 (biological process, cellular component, and molecular function), and C6 (oncological signature) (Subramanian et al. 2005). A *p* value < 0.05 was considered statistically significant, and *q* < 0.25 was the false discovery rate.

### Statistical analysis

R software v3.6.1 (R Foundation for Statistical Computing, Vienna, Austria), SPSS version 25 (SPSS Inc.), and GraphPad Prism v8.0 (GraphPad Software Inc., the USA) were used for statistical analysis. The Fisher’s exact test or Pearson χ^2^ test was used to assess for qualitative variables and *t*-test for quantitative variables. A *p* value < 0.05 was considered statistically significant.

## Results

### Identification of DEGs

**Figure 1.**
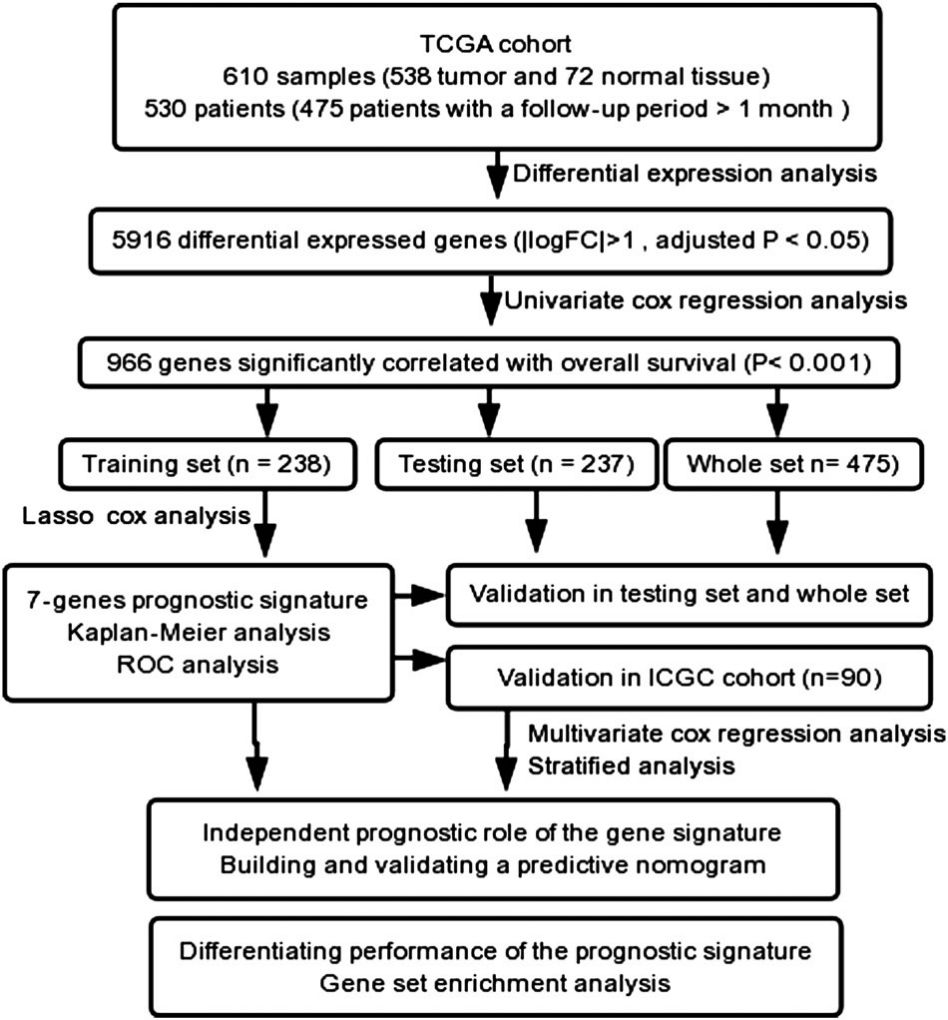
Study flow.

The flow diagram used in this study is presented in Figure 1. We identified 5916 DEGs when comparing the KIRC samples (*n *= 538) and normal tissues (*n *= 72), including 3865 up-regulated genes and 2051 down-regulated genes (Supplementary File 1: Fig. S1).

### Construction of the seven-gene prognostic signature

**Figure 2.**
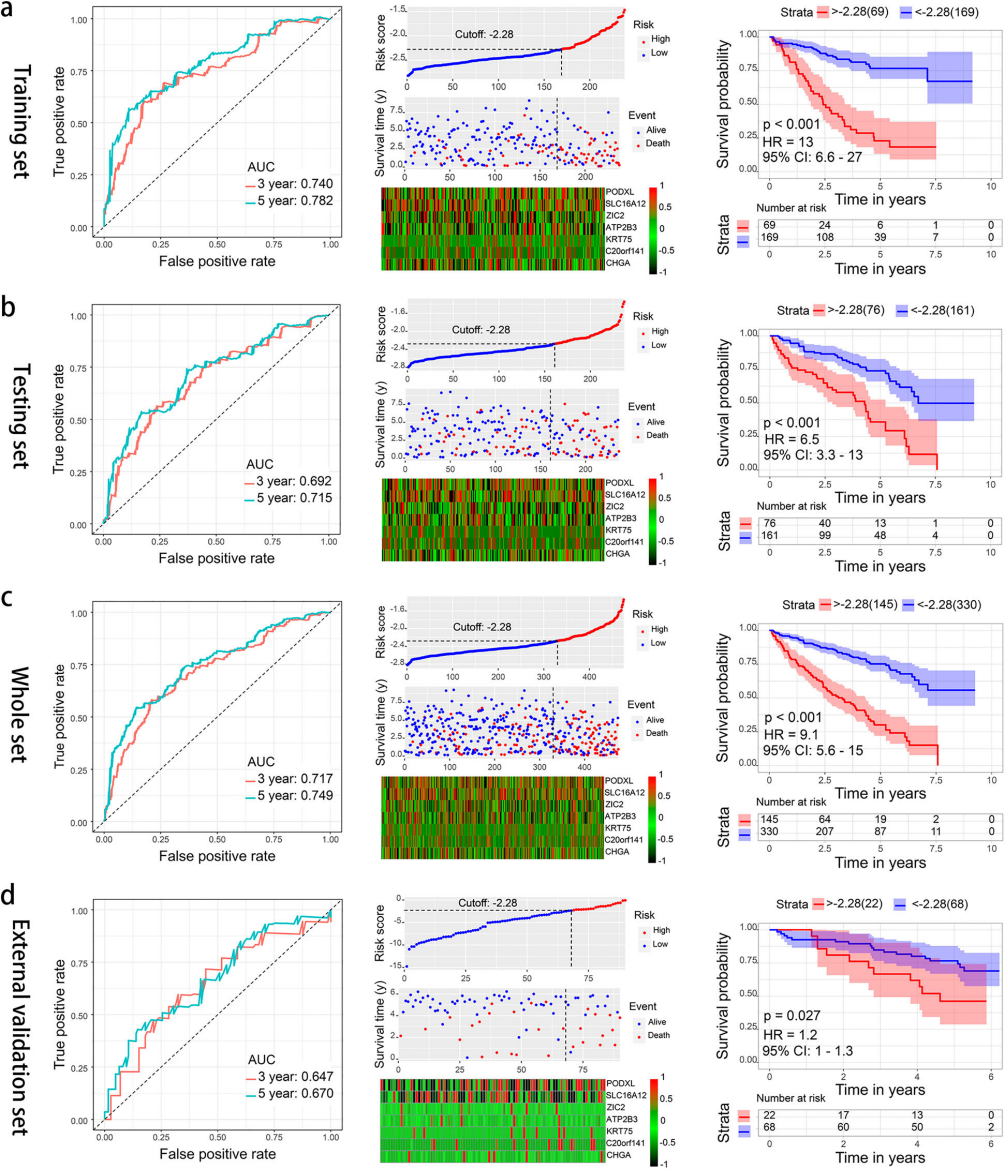
Time-dependent ROC analysis, risk score analysis (risk score and heatmap of mRNA expression), and Kaplan–Meier analysis for the seven-gene signature in the training set of the TCGA cohort (a), the testing set of the TCGA cohort (b), the whole set of the TCGA cohort (c), and the set of the ICGC cohort (d). ROC, receiver operating characteristic; TCGA, The Cancer Genome Atlas; ICGC, International Cancer Genome Consortium.

In total, 475 patients with a follow-up period longer than 1 month were randomly divided into training set (n = 238) and testing set (*n* = 237). There was not significant difference in the clinical characteristic between the groups (Table 1). Moreover, 966 genes significantly correlated to the OS of KIRC were identified via a univariate Cox regression analysis. Subsequently, lasso penalized Cox regression analysis was conducted using the training set (Supplementary File 2: Fig. S2). Seven genes were identified and used to establish a prognostic gene signature. The genes were podocalyxin-like (PODXL), solute carrier family 16 member 12 (SLC16A12), Zic family member 2 (ZIC2), ATPase plasma membrane Ca2+ transporting 3 (ATP2B3), keratin 75 (KRT75), chromosome 20 open reading frame 141 (C20orf141), and chromogranin A (CHGA). The risk score = −0.1696 * Expression_PODXL_ −0.0104 * Expression_SLC16A12_ + 0.0398 * Expression_ZIC2_ + 0.0057 * Expression_ATP2B3_ + 0.0035 * Expression_KRT75_ + 0.0186 * Expression_C20orf141_ + 0.0045 * Expression_CHGA_. According to the optimal cutoff value of the risk score, the patients were classified into the high- and low-risk groups. The areas under the receiver operating characteristic curve (AUCs) for the 3- and 5-year OS were 0.740, 0.782; 0.692, 0.715; and 0.717, 0.749 for the training set, testing set, and whole set, respectively. The Kaplan–Meier curve showed that the patients in the high-risk group presented with a significantly poorer OS than the patients in the low-risk group (all *p* < 0.001) (Figure 2a–c). In addition, our seven-gene signature had a better C-index and AUC for 3- and 5-year OS prediction compared with the other five reported gene signatures (Supplementary File 3: Fig. S3, Supplementary File 4: Table S1). The results indicated that our seven-gene signature had a good performance in predicting the survival of patients with KIRC.

**Table 1.** Clinical features of KIRC patients in training set, testing set and whole set

Clinical features	Whole set	Training set	Testing set	*p*
Total	*n *= 475	*n *= 238	*n *= 237	
Dead (%)	152 (32.0)	70 (29.4)	82 (34.6)	0.226
Mean age (SD), years	60.4 (12.0)	60.9 (12.3)	59.9 (11.9)	0.377
Female (%)	161 (33.9)	84 (35.3)	77 (32.5)	0.518
Tumor grade				0.982
G1	8 (1.7)	4 (1.7)	4 (1.7)	
G2	200 (42.1)	98 (41.2)	102 (43.0)	
G3	196 (41.3)	100 (42.0)	96 (40.5)	
G4	71 (14.9)	36 (15.1)	35 (14.8)	
TNM stage				0.965
Stage I	235 (49.5)	117 (49.2)	118 (49.8)	
Stage II	50 (10.5)	26 (10.9)	24 (10.1)	
Stage III	109 (22.9)	56 (23.5)	53 (22.4)	
Stage IV	81 (17.1)	39 (16.4)	42 (17.7)	

**Abbreviations:** KIRC: kidney renal clear cell carcinoma; TNM: tumor-node-metastasis.

### External validation of the prognostic gene signature and gene change

External validation was conducted in the ICGC cohort with the same method mentioned above. In total, 90 patients with a follow-up period longer than 1 month were divided into the high- and low-risk groups with the same cutoff value. The AUC for the 3- and 5-year OS were 0.647 and 0.670, respectively. The OS was significantly poorer in the high-risk group than in the low-risk group (*p* = 0.027) (Figure 2d).

**Figure 3.**
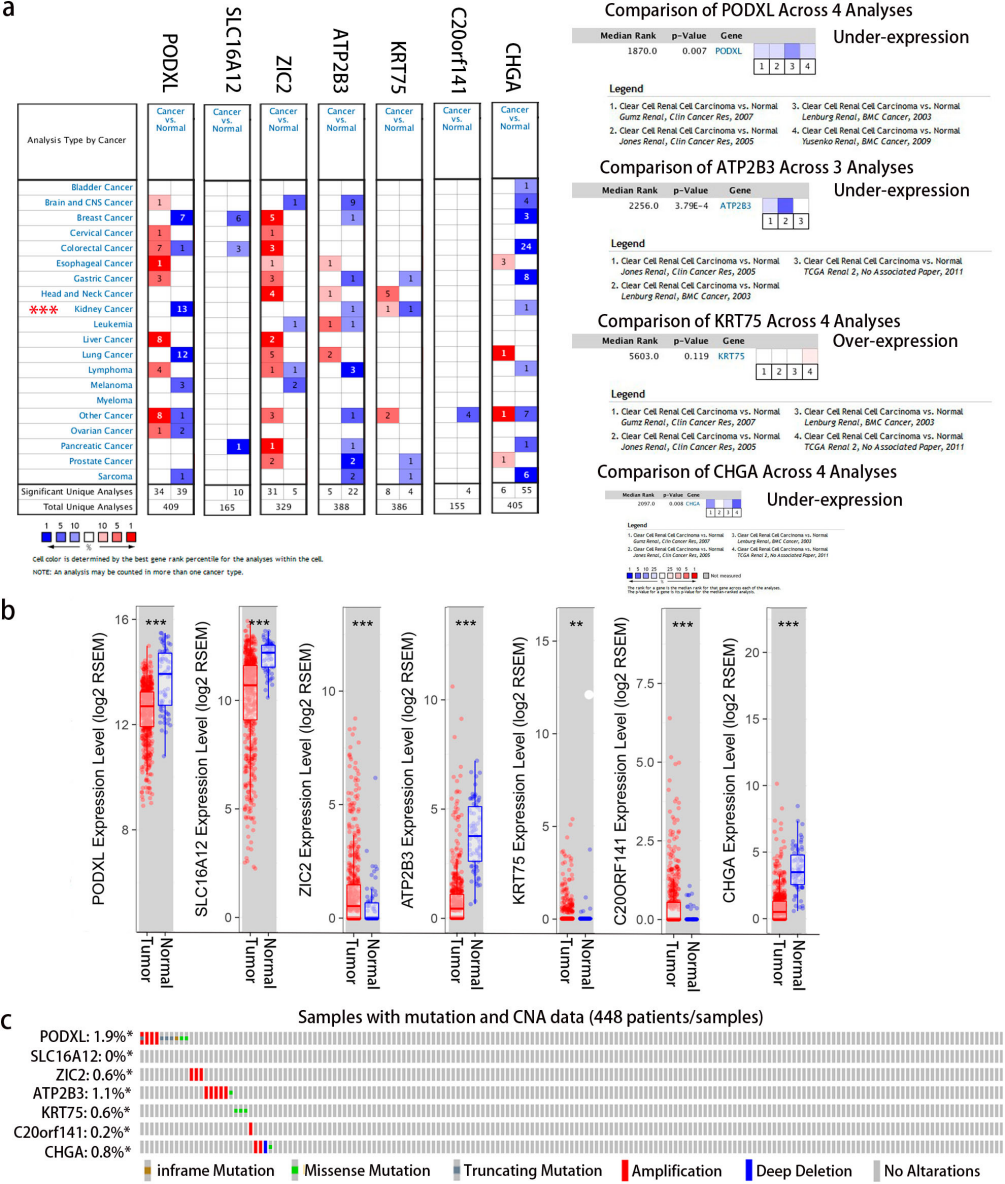
Expression and genetic alterations of the seven-gene signature. (a) The expression of the seven genes using data from the Oncomine database (https://www.oncomine.org/). The database had missing data of SLC16A12, ZIC2, and C20orf141 in KIRC. (b) The expression of the seven genes using data from the TIMER database (https://cistrome.shinyapps.io/timer/). (c) Genetic alterations of the seven genes based on data from the TCGA (http://www.cbioportal.org/).

Furthermore, we validated the expression of the seven genes in the prognostic signature using data from several online databases. Consistent with the result in our study, PODXL, ATP2B3, and CHGA were significantly underexpressed. Meanwhile, KRT75 was significantly overexpressed in KIRC using information from both the Oncomine and TIMER databases (Figure 3a, b). Although the data in the Oncomine database were lacking, SLC16A12 was significantly underexpressed, whereas ZIC2 and C20orf141 were significantly overexpressed in KIRC based on data from the TIMER database. The cBioportal for Cancer Genomics database was assessed, and it showed that 5.2% of 488 patients had genetic alterations in the seven genes. Amplification was the most commonly observed change (Figure 3c). Thus, the aberrant expression of the seven genes in the prognostic signature was validated, which could be partly explained by the genetic alteration.

### Independent prognostic role of the gene signature

**Figure 4.**
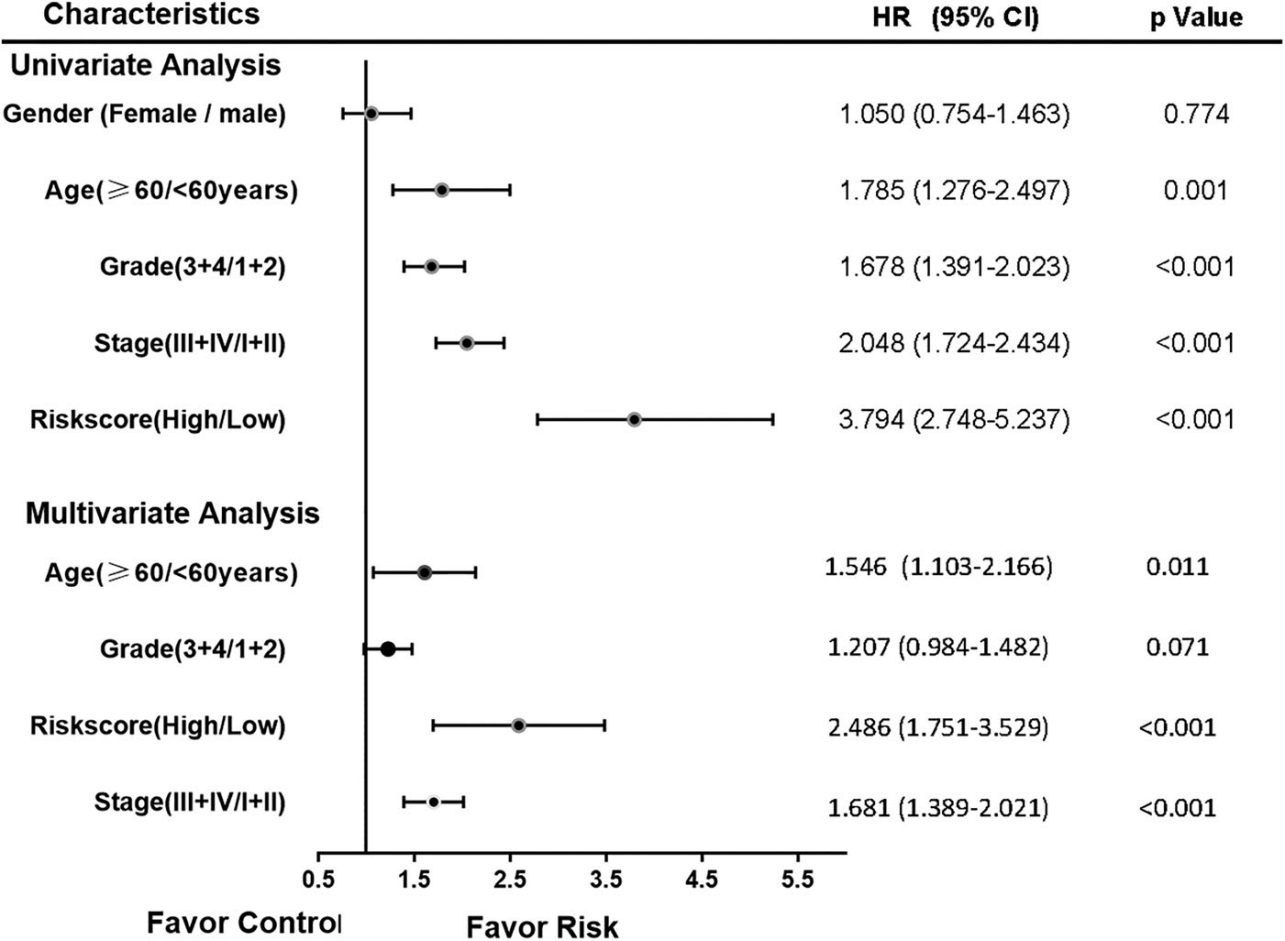
Forrest plot of the univariate and multivariate Cox regression analyses of KIRC.

**Figure 5.**
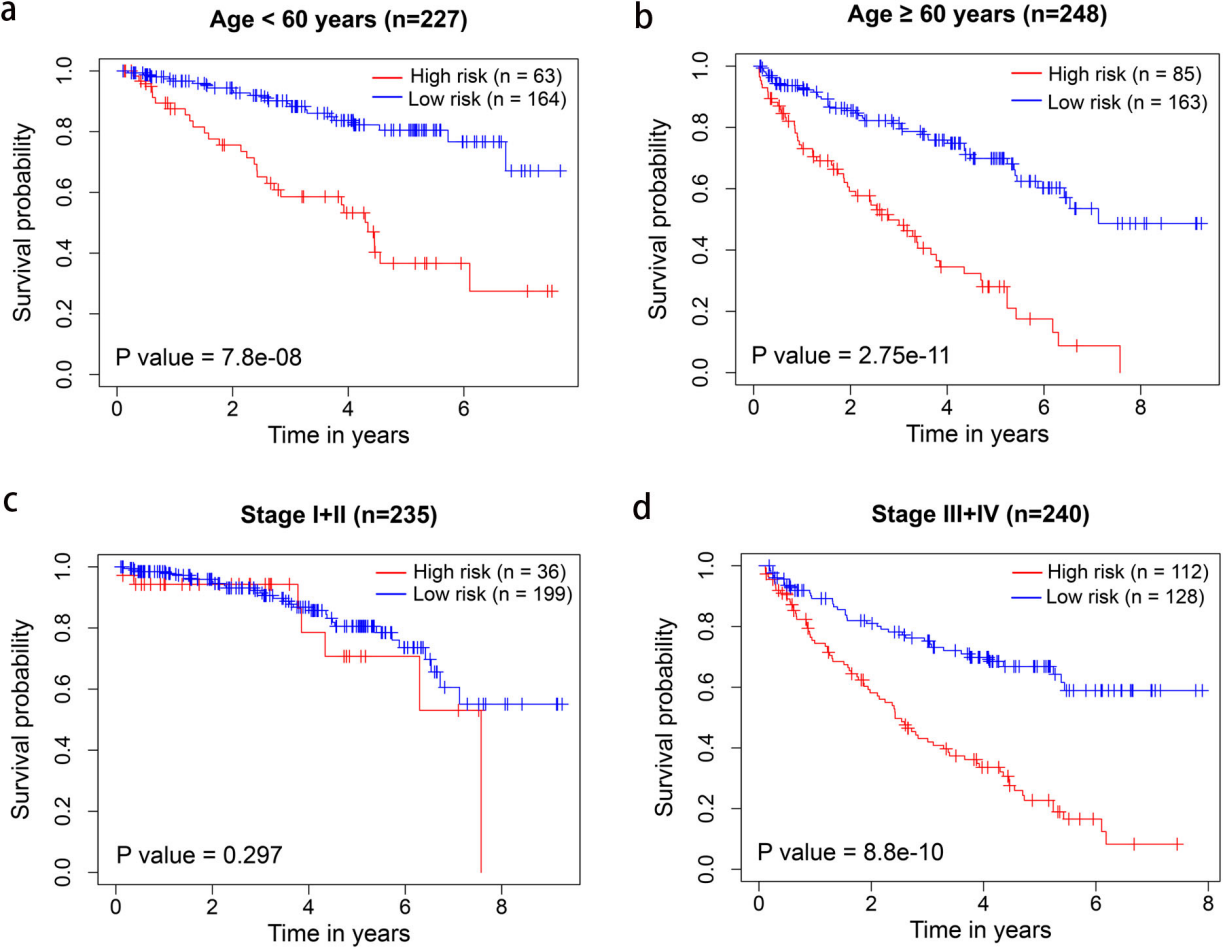
Kaplan–Meier survival analysis of the seven-gene signature using the TCGA cohort according to age and TNM stage stratification. (a) Age < 60 years; (b) age ≥ 60 years; (c) stage I + II; (d) stage III + IV.

Univariate and multivariate Cox regression analyses were performed in 475 patients with complete clinical data, including age, gender, TNM stage, and grade. Subsequently, age, TNM stage, and risk score were considered as independent prognosis factors of OS (Figure 4). Moreover, stratified analysis was performed according to age and TNM stage. The OS was significantly poorer in the high-risk group than in the low-risk group both in age < 60 and≥ 60 years (Figure 5a, b). However, the survival between the high- and low-risk groups significantly differed only in stage III/IV, but not in stage I/II (Figure 5c, d).

### Establishing and validating a predictive nomogram

**Figure 6.**
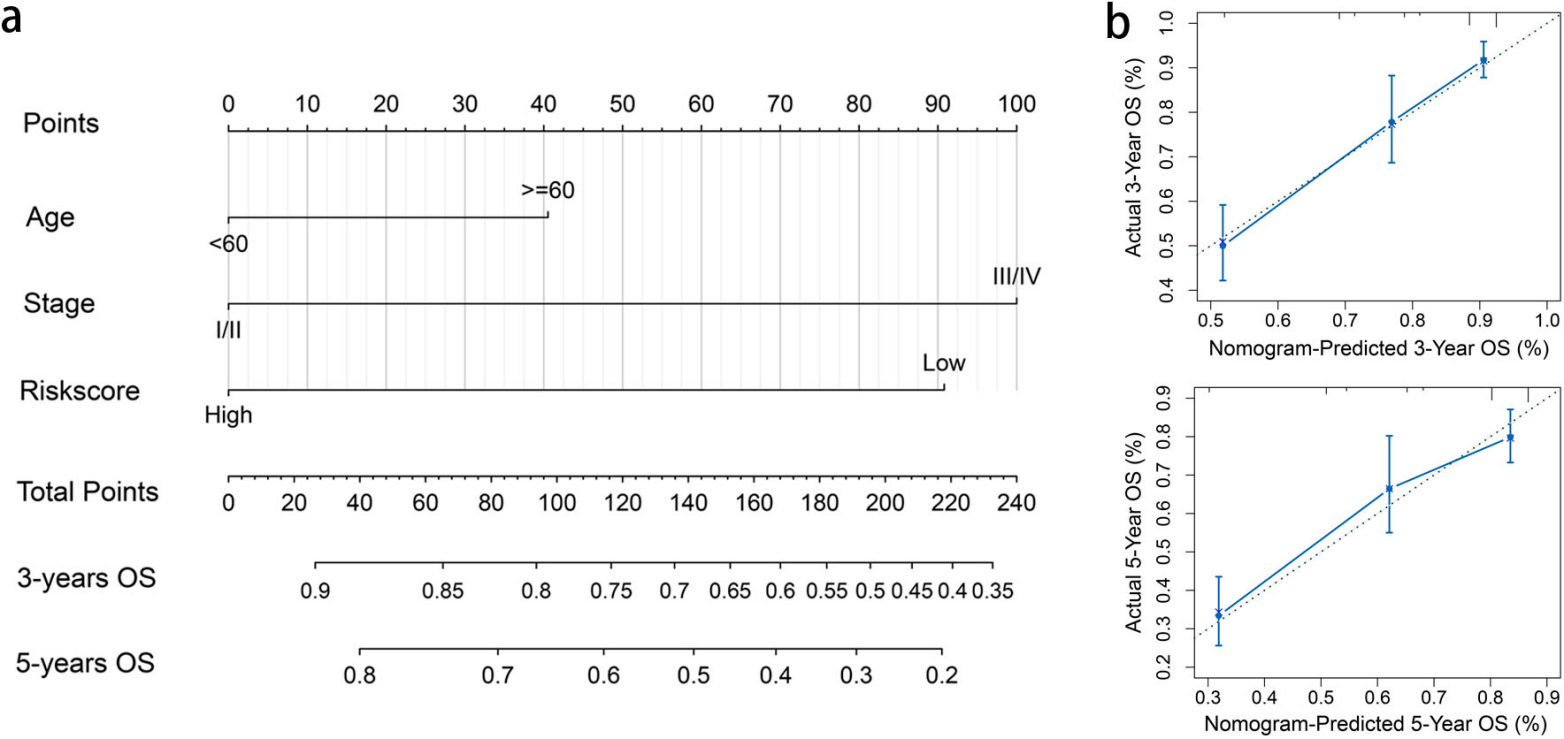
Nomogram for predicting the overall survival of KIRC patients. (a) The nomogram plot was based on three independent prognostic factors of KIRC. (b) Calibration plot for the internal validation of the nomogram.

**Figure 7.**
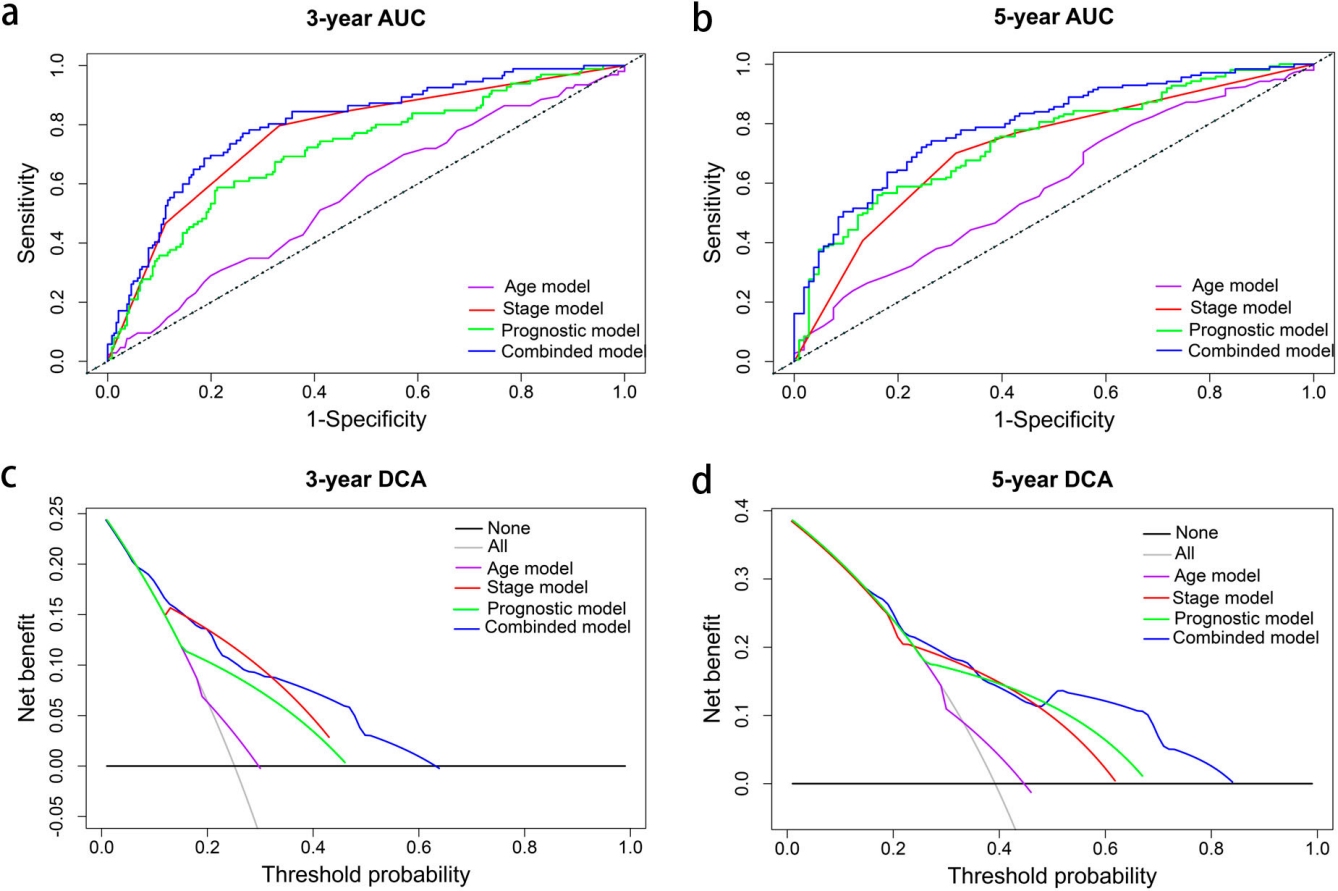
The time-dependent ROC and DCA curves of the nomogram. (a, b) The time-dependent ROC curves of the nomogram compared with other independent prognostic factors for the 3- and 5-year overall survival of patients with KIRC. (c, d) The DCA curves of the nomograms compared with other independent prognostic factors of the 3- and 5-year overall survival of patients with KIRC.

A nomogram was established, and three independent prognostic factors (age, stage, and risk score) were included (Figure 6a). The calibration plot showed that the nomogram is effective in predicting the 3- and 5-year OS (Figure 6b). The C-index values were 0.60, 0.74, 0.70, and 0.78 for the age, stage, prognostic, and combined models, respectively. The AUCs were 0.80 for 3-year OS and 0.79 for 5-year OS (Table 2). When combining our prognostic model with age and stage, the AUC increased both for the 3- and 5-year OS, and the net benefit also improved for patients with KIRC (Figure 7).

**Table 2.** Comparison of the nomogram (combined model) with age, TNM stage and prognostic model

Models	3-year AUC (95%CI)	*P*-value	5-year AUC (95%CI)	*P*-value
Age model	0.57 (0.50–0.63)		0.59 (0.52–0.67)	
TNM stage model	0.77 (0.71–0.82)		0.72 (0.65–0.79)	
Prognostic model	0.71 (0.65–0.78)		0.75 (0.68–0.81)	
Nomogram (combined) model	0.80 (0.75–0.85)		0.79 (0.74–0.85)	
Nomogram vs. age model		<0.001		<0.001
Nomogram vs. stage model		<0.04		<0.001
Nomogram vs. prognostic model		<0.001		0.054

AUC area under curve, CI confidence interval, TNM tumor-node-metastasis.

### Identifying the capacity of the prognostic gene signature

**Figure 8.**
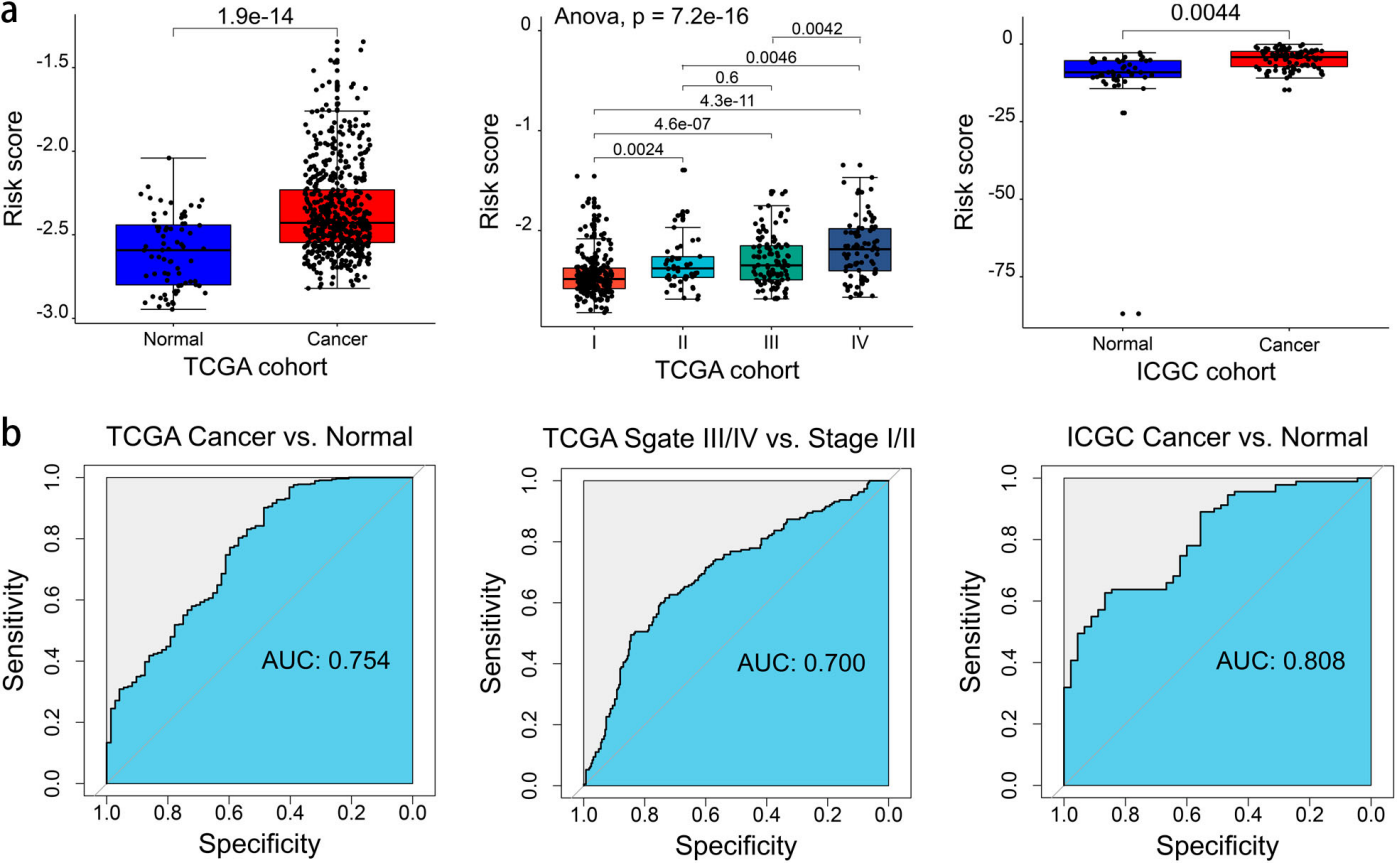
Capacity of the seven-gene signature in differentiating KIRC from normal tissues. (a) The difference in the risk score between the groups or TNM stage. (b) The ROC curve of the risk score between the groups or TNM stage.

We explored the capacity of the gene signature to distinguish KIRC from normal tissues, and results showed that the risk score was significantly higher in KIRC tissues than in normal tissues. In addition, the risk score significantly increased with stage progression (Figure 8a). The AUC indicated a modest diagnostic ability for KIRC (Figure 8b). These results showed a potential function of the gene signature in the differential diagnosis of KIRC.

### Gene set enrichment analyses

GSEA were performed on 475 patients from the TCGA cohort. In total, 3 hallmark gene sets, 2 KEGG pathways, 79 GO terms, and 2 oncological signatures were enriched. Both oncological signatures (Rb-P107 and CSR-LATE) were enriched in the high-risk group and none in the low-risk group (Supplementary File 5: Table S2).

## Discussion

In recent years, the incidence and mortality of kidney cancer has been increasing, and the treatment of kidney cancer remains a significant challenge worldwide because of its poor prognosis (Gray and Harris 2019; Siegel et al. 2019). Thus, patients with poor survival must be identified. Although several prognostic factors have been reported, TNM stage is still the most valuable predictor of KIRC (Frank et al. 2002). However, the clinical outcomes may differ significantly among patients with the same TNM stage due to the heterogeneity of KIRC (Martínez-Salamanca et al. 2011; Park et al. 2019). Therefore, new reliable prognostic biomarkers are urgently needed to establish more accurate prognostic models. Recently, mRNA gene signatures have been considered as the potential predictors of prognosis for KIRC (Chen et al. 2019; Wu et al. 2019; Zhang et al. 2019).

In our study, we established a seven-gene signature (including PODXL, SLC16A12, ZIC2, ATP2B3, KRT75, C20orf141, and CHGA) for the prognostic prediction for KIRC, and the efficiency was good both in the TCGA and ICGC cohorts. In addition, it was comparable with the other five models (Zhan et al. 2015; Chen et al. 2019; Hu et al. 2019; Wu et al. 2019; Zeng et al. 2019). The risk score calculated using the seven-gene signature was an independent prognostic factor for KIRC, and it was effective in stratifying the OS of patients. The ROC and DCA showed that the nomogram combining the seven-gene signature and other clinical prognostic factors is efficient in predicting the survival of patients with KIRC. However, patients in the high-risk group had a significantly poorer OS only in stage III/IV, but not in stage I/II. This result might be attributed to the the small sample size of the high-risk group (*n* = 36) and the relatively low clinical progression of stage I and II disease. Thus, the difference became challenging to distinguish. Another interesting point was that the seven-gene signature had a modest ability to differentiate KIRC from normal tissues, and this result indicated that the signature had a potential role in the differential diagnosis of KIRC. Finally, GSEA revealed two oncological signatures in the high-risk group, which might partially explain the potential molecular mechanisms.

Most studies mainly focused on cancer-related genes. However, some genes in our signature were not involved in cancer. PODXL is an anti-adhesive transmembrane glycoprotein, which is a member of the CD34 family (Snyder et al. 2015). Several studies showed that PODXL is associated with the invasion, migration, epithelial–mesenchymal transition, and metastasis of cancers, and it could be considered an independent prognostic factor (Meng et al. 2011; Lin et al. 2014; Taniuchi et al. 2016). However, only few studies have reported the role of PODXL in KIRC. SLC16A12 is a member of the SLC16A family, and its function is not known. A recent study showed that SLC16A12 can be a prognostic factor for patients in KIRC, indicating that SLC16A12 might be a critical tumor suppressor (Mei et al. 2019). ZIC2 is a member of the ZIC family and is a transcription factor (Inaguma et al. 2015). Some studies revealed that ZIC2 could regulate the progression of several types of cancer, including nasopharyngeal carcinoma (Shen et al. 2017), bladder cancer (Wang et al. 2017), and liver cancer (Zhu et al. 2015). However, the functions of ZIC2 in KIRC are not fully elucidated. ATP2B3 belongs to the family of P-type primary ion transport ATPases, and it is highly expressed in the cerebellum and brain. ATP2B3 plays an important role in the regulation of neuronal Ca2 + . However, there are limited data about its role in cancer (Cali et al. 2015). KTR75 is a member of the type II keratin family, and it plays an essential role in nail and hair formation (Duverger et al. 2016). Its other roles in cancer remain unknown. The function of C20orf141, a protein-coding gene, is still not fully elucidated to date. CHGA is a protein in the secretory granules of several normal and neoplastic neuroendocrine cells, and it has been considered an important biomarker of neuroendocrine neoplasms (Oberg 1997; Tomassetti et al. 2001; Mahapatra et al. 2005). In addition, its use as an early diagnosis biomarker for several types of cancer, including prostate cancer and gastric cancer, has been approved (Yang and Chung 2008; Ma et al. 2010). However, no study has assessed the role of CHGA in KIRC.

In our study, the seven-gene signature was based on mRNA expression instead of methylation status or somatic mutations, and it could be more cost-effective when used in clinical practice. In addition, the gene signature was more reliable because it was validated using data from the ICGC cohort and those from other online databases. Although our gene signature had a good performance in predicting the survival of patients with KIRC, it still had several limitations. First, the seven-gene signature was generated base on data from TCGA, in which most patients were White and Asian. Second, the signature was validated using data from the ICGC, and the sample size was still limited. Thus, multiple centers across different populations are important for the further validation of our model. Third, precise and rigorous basic experiments must be conducted to identify the biological functions of signature genes. Finally, since our seven-gene signature could distinguish KIRC from normal tissues, its ability to classify the different types of kidney cancer should be explored.

## Conclusion

our study developed a novel seven-gene signature and nomogram for predicting the OS of patients with KIRC, which might be helpful for clinicians in establishing individualized treatments.
